# Identification of mesothelioma-specific sialylated epitope recognized with monoclonal antibody SKM9-2 in a mucin-like membrane protein HEG1

**DOI:** 10.1038/s41598-018-32534-8

**Published:** 2018-09-24

**Authors:** Rieko Matsuura, Hiroyuki Kaji, Azusa Tomioka, Takashi Sato, Hisashi Narimatsu, Yasuhiro Moriwaki, Hidemi Misawa, Kohzoh Imai, Shoutaro Tsuji

**Affiliations:** 1 Kanagawa Cancer Center Research Institute, Yokohama, Japan; 2 Glycoscience & Glycotechnology Research Group, Biotechnology Research Institute for Drug Discovery, National Institute of Advanced Industrial Science and Technology, Tsukuba, Japan; 3 Division of Pharmacology, Faculty of Pharmacy, Keio University, Tokyo, Japan; 4 Institute of Medical Science, University of Tokyo, Tokyo, Japan

## Abstract

The anti-mesothelioma mAb SKM9-2 recognizes the sialylated protein HEG homolog 1 (HEG1). HEG1 is a 400 kDa mucin-like membrane protein found on mesothelioma. SKM9-2 can detect mesothelioma more specifically and sensitively than other antibodies against current mesothelioma markers; therefore, SKM9-2 would be likely useful for the precise detection and diagnosis of malignant mesothelioma. In the present study, we investigated the epitope of SKM9-2. We analyzed the binding of SKM9-2 to truncated HEG1 and candidate epitope-fused glycosylphosphatidylinositol-anchor proteins. The epitope of SKM9-2 was identified as an *O*-glycosylated region, 893-SKSPSLVSLPT-903, in HEG1. An alanine scanning assay of the epitope showed that SKM9-2 bound to a simple epitope in HEG1, and the SKxPSxVS sequence within the epitope was essential for SKM9-2 recognition. Mass spectrometry analysis and lectin binding analysis of soluble epitope peptides indicated that the SKM9-2 epitope, in which Ser^897^ was not glycosylated, contained two disialylated core 1 *O*-linked glycan-modified serine residues, Ser^893^ and Ser^900^. Neuraminidase treatment analysis also confirmed that the epitope in mesothelioma cells contained a similar glycan modification. The specific detection of mesothelioma with SKM9-2 can thus be performed by the recognition of sialylated glycan modification in the specific region of HEG1.

## Introduction

A glycoprotein containing many *O*-linked glycans, such as those of the mucin family and a mucin-like protein, can be a good cancer-related marker^1–3^. Such glycoproteins are associated with tumor-specific glycosylation, such as the alteration in the core type^4,5^, decrease in glycan-attachment^6,7^, or formation of immature or irregular glycans^8,9^. The detection of these glycan-related changes using a monoclonal antibody (mAb) can contribute to the reliable finding of tumor. Therefore, mAbs against glycan-related changes of the mucin or mucin-like proteins have been used for detection or monitoring of tumors in serologic diagnoses^10,11^. In addition, a mAb against the glycan-related change in a specific mucin-like protein can be used to distinguish the malignancy of tumors via immunohistochemistry^12^. The reactivity of mAb that recognizes the glycan-related change does not correspond to gene expression of the antigen. Due to this, the verification of results using another mAb is often difficult. Therefore, epitope analysis containing the altered glycan is important for clarifying the tumor-specific difference recognized by the mAb and ensuring the reliability of the observation for investigators.

Malignant mesothelioma is a fatal tumor caused by exposure to asbestos^13^. The absence of mesothelioma-specific markers has often made it difficult to diagnose malignant mesothelioma^14^. In our previous study^15^, we identified the sialylated protein HEG homolog 1 (HEG1) as a novel and precise mesothelioma marker. HEG1 was first reported as the encoded product of the *heart of glass* gene regulating the concentric growth of zebrafish hearts^16^. The mouse *HEG1* gene has been linked to cardiovascular organ development^17^. Human HEG1 on mesothelioma cells is a 400 kDa mucin-like membrane protein with a heavily *O*-glycosylated Ser/Thr rich region that accounts for ~70% of the molecule, but does not contain tandem repeat sequences^15^. HEG1 is expressed on apical cell surfaces and is associated with mesothelioma cell proliferation^15^. We have shown in the previous study that the anti-HEG1 mAb SKM9-2 recognizes the glycopeptide sequence and can detect mesothelioma more specifically (99%) and sensitively (92%) than other mAbs against current mesothelioma markers. Therefore, SKM9-2 may be useful for the precise detection and diagnosis of malignant mesothelioma^15^.

In the present study, we investigated the epitope of mAb SKM9-2. We identified that SKM9-2 recognizes a single epitope on HEG1–893-SKSPSLVSLPT-903. Further, the epitope, in which Ser^897^ was not glycosylated, contained two disialylated core 1 *O*-linked glycan (disialyl T)-modified serine residues, Ser^893^ and Ser^900^. The binding of SKM9-2 required not only the amino acid sequences but also the attachment of sialylated glycans. SKM9-2’s recognition of glycan modification in this specific region of HEG1 could be useful for the precise detection and diagnosis of malignant mesothelioma.

## Results

### Epitope of mAb SKM9-2

**Figure 1:**
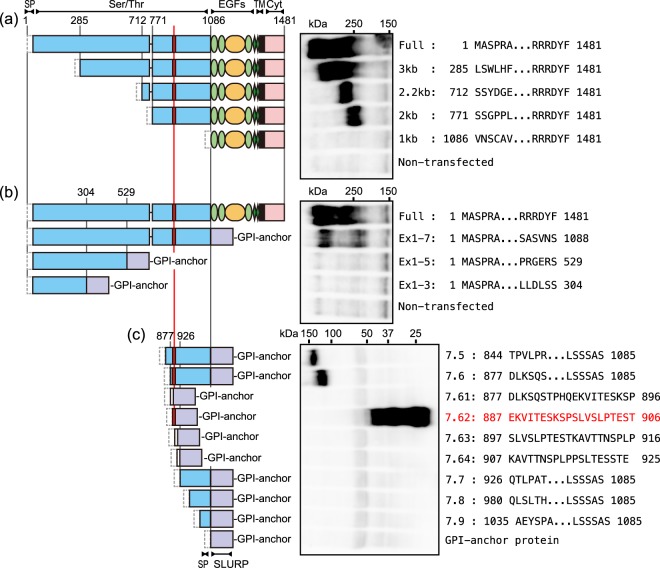
SKM9-2 binding to truncated mutants of HEG1. Schematics of full-length HEG1 (Full) and the truncated mutants are indicated on the left side and the results of western blotting using SKM9-2 are shown on the right side. Truncated amino acid position, which is based on a full-length variant of HEG1 containing exon 6, is shown as a number in the schematic of domain structure. The region of the SKM9-2 epitope is shown as a red square. Each mutant had a signal peptide (dashed line square). SLURP GPI-anchor protein^18^ was used as a fused GPI-anchor protein (violet square). The terminal sequences of the HEG1 peptide are shown in the western blot lane. The samples were resolved by 6% SDS-PAGE (**a** and **b**) or 4–15% SDS-PAGE (**c**) under reducing conditions and were detected by western blotting using SKM9-2. Cyt, cytoplasmic domain; EGFs, EGF related domains; Ser/Thr, serine/threonine rich region; SLURP, secreted lymphocyte antigen-6/urokinase-type plasminogen activator receptor-related peptide; SP, signal peptide; TM, transmembrane domain. Full-length blots are presented in Supplementary Fig. S7.

**Figure 2:**
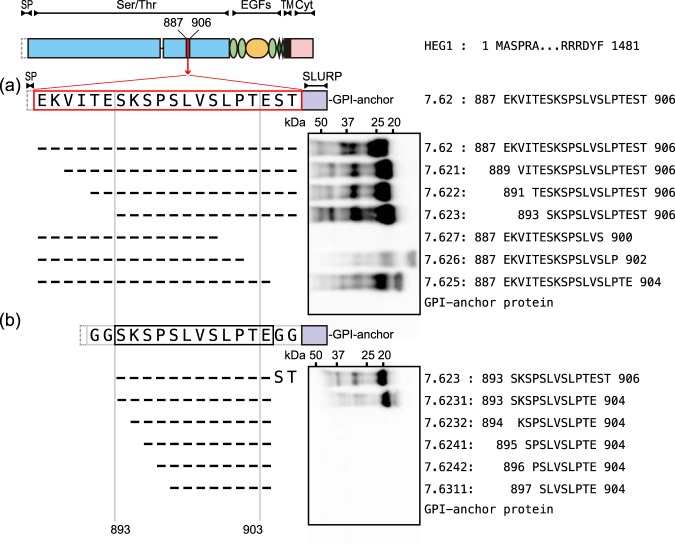
Epitope mapping of SKM9-2. Schematic of epitope peptides (left side) and western blots resolved by 4–15% SDS-PAGE (right side) are shown. The HEG1 peptide sequences are indicated in the western blot lane. Each epitope peptide was fused with a signal peptide (dashed line square) and a SLURP GPI-anchor protein (violet square). Full-length blots are presented in Supplementary Fig. S8.

**Figure 3:**
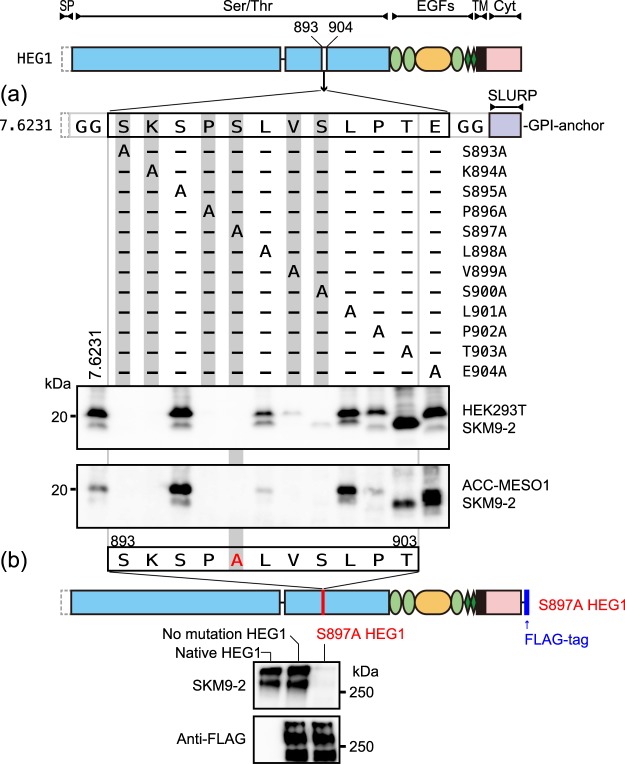
Alanine scanning of SKM9-2 epitope. (**a**) Schematic shows the position of Ala substitution in the epitope-fused GPI-anchor protein (7.6231). Cell lysates of transfected HEK293T (upper panel) or transfected ACC-MESO1 (lower panel) were resolved by 4–15% SDS-PAGE and analyzed by western blotting using SKM9-2. (**b**) S897A mutant of FLAG-tagged HEG1 in HEK293T was resolved by 4–15% SDS-PAGE and analyzed by western blotting using SKM9-2 (upper panel) or anti-FLAG mAb (lower panel). Native HEG1 in ACC-MESO4 and FLAG-tagged HEG1 without mutation in HEK293T were used as controls. Full-length blots are presented in Supplementary Fig. S9.

As previously described, mAb SKM9-2 recognizes an extracellular region of the sialylated HEG1^15^. The SKM9-2 epitope was investigated using HEG1 deletion mutants transfected in HEK293T cells. Since the tested mutants had a signal peptide and a transmembrane domain, they were expressed as type I membrane proteins modified with many glycans, such as the full-length HEG1. As shown in Fig. 1a, mutants deleted up to Ser^770^ (named as 3 kb, 2.2 kb, and 2 kb) were recognized by SKM9-2 upon western blot analysis, whereas a mutant without the region from Met^1^ to Ser^1085^ (1 kb) was not detected with SKM9-2. For the analysis of the *C*-terminal deletion, we used proteins that were fused with a glycosylphosphatidylinositol (GPI)-anchor protein^18^ to compensate for the loss of the transmembrane domain. A fused protein including most of the extracellular domain, which was deleted from Cys^1089^ to Phe^1481^ in HEG1, was recognized by SKM9-2 (named as Ex1–7), but proteins without the exon 7 region, deleted from Ser^305^ (Ex1–3) or Ile^530^ (Ex1–5), were not detected (Fig. 1b). In the following experiments, a short HEG1 peptide fused with a signal peptide and the GPI-anchor protein was used (Fig. 1c). Two fused proteins including the region from Asp^877^ to Ser^1085^ (named as 7.5 and 7.6) were recognized by SKM9-2, but proteins with sequences beginning after Gln^926^ were not detected (7.7–7.9). Therefore, the epitope of SKM9-2 was confined to the region from Asp^877^ to Glu^925^. In the assay with fused proteins, including overlapping peptides (named as 7.61–7.64), only a protein fused with the region from Glu^887^ to Thr^906^ (7.62) was clearly detected by SKM9-2. Thus, this region contains the SKM9-2 epitope. In Fig. 1a,b, SKM9-2-recognized bands were detected as broad bands. This heterogeneity can potentially be caused by several types of *N*-linked and *O*-linked glycans in the Ser/Thr rich region. The multiple bands of peptide-fused GPI-anchor proteins (Fig. 1c, Figs 2, 3) may be caused by different sizes of *O*-linked glycans in the epitope peptide, since the peptide-fused protein does not have an *N*-glycosylation site.

To identify the minimum recognition site of SKM9-2, the amino acid sequences of 7.62 were further truncated. As shown in Fig. 2a, truncation up to Glu^892^ on the *N*-terminal side did not affect SKM9-2 reactivity (7.62, and 7.621–7.623); whereas, the truncation on *C*-terminal side decreased SKM9-2 reactivity. The Thr^903^-deleted mutant (7.626) was weakly recognized by SKM9-2 and a shorter peptide (7.627) was not bound (Fig. 2a). In the next experiment (Fig. 2b), Gly-Gly spacers were added to both ends of a candidate peptide to avoid unexpected structural change by the neighboring residues. Although the peptide including Ser^893^ was bound by SKM9-2 (7.6231), the shorter peptides without Ser^893^ were not recognized by SKM9-2 (7.6232, 7.6241, 7.6242, and 7.6311). These results suggest that the epitope region of SKM9-2 is 893-SKSPSLVSLPT-903.

Further, we determined the important amino acid residues responsible for SKM9-2 binding by alanine scanning of 7.6231. As shown in Fig. 3a, alanine substitution for Ser^893^, Lys^894^, Pro^896^, Ser^897^, Val^899^, or Ser^900^ resulted in the loss of recognition by SKM9-2. The replacement of Glu^904^ did not affect SKM9-2 binding. The alanine-substituted mutant of Thr^903^ in the presence of Glu^904^ was recognized by SKM9-2, unlike the results of Thr^903^ deletion (7.626) in Fig. 2a; however, its molecular size was smaller than that of the other alanine mutants. Similar results using alanine scanning were also observed in transfectants using the mesothelioma cell line ACC-MESO1 (Fig. 3a lower panel). The full-length HEG1 with a single mutation (S897A) was not recognized by SKM9-2, though its expression was confirmed through the detection of the FLAG epitope (Fig. 3b). Taken together, these results suggest that SKM9-2 binds to the simple epitope (893-SKSPSLVSLPT-903) in HEG1, and SKxPSxVS in this sequence is essential for recognition by SKM9-2.

### *O*-linked glycan in SKM9-2 epitope

**Figure 4:**
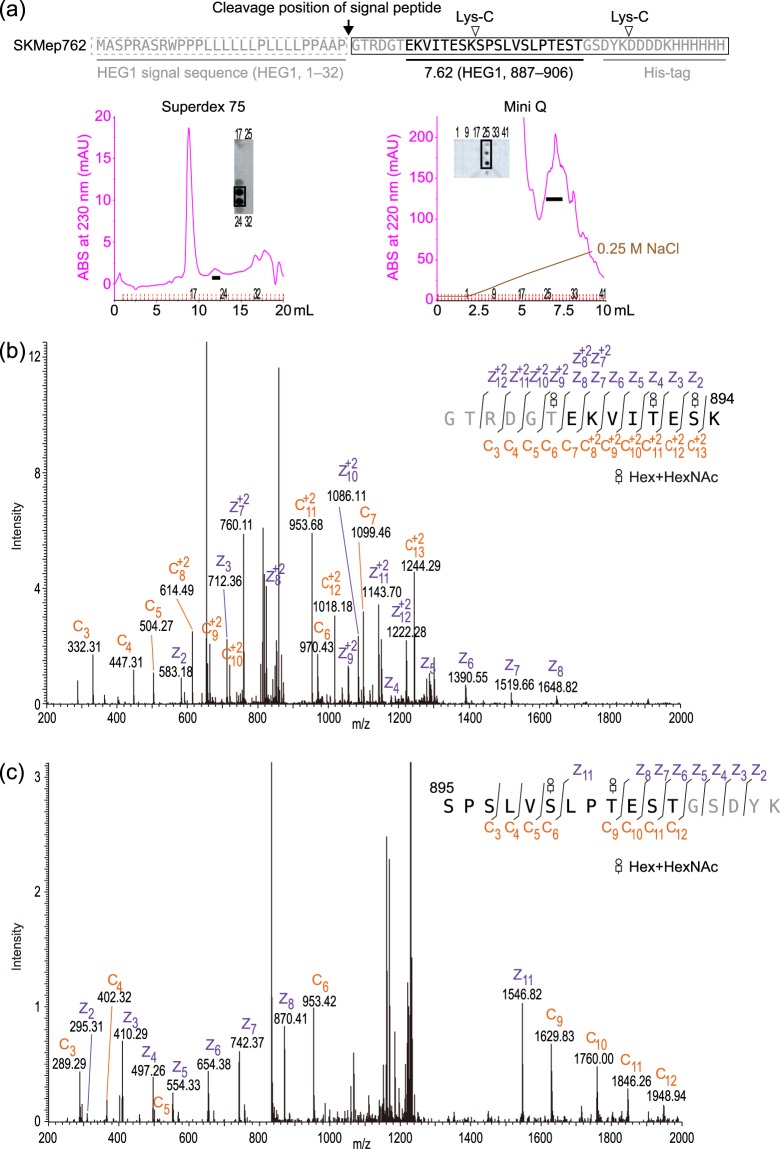
MS analysis of soluble SKMep762. (**a**) Purification of soluble SKMep762. Sequences of SKMep762 are shown at the top. The secreted SKMep762 was purified using HisTrap excel, and then separated by Superdex 75 10/300 GL (left chromatogram) and Mini Q 4.6/50 PE (right chromatogram). Dot blots of fractions using SKM9-2 were also shown. Pooled fractions are indicated as a black bar. (**b**) LC-MS spectrum of *N*-terminal glycopeptide ion. Purified SKMep762 was treated by Lys-C-digestion and desialylation, and analyzed by LC-MS (ETD). An MS/MS spectrum of a major component (*m/z* = 2616.19) in total ion current is shown. (**c**) LC-MS spectrum of *C*-terminal glycopeptide ion. MS/MS spectrum of another major component (*m/z* = 2498.13) in total ion current is shown. Hex, hexose; HexNAc, *N*-acetylhexosamine.

The SKM9-2 binding requires sialylation^15^ and the SKM9-2 epitope had no *N*-glycosylation site as mentioned before; thus, we hypothesized that the SKM9-2 epitope would contain *O*-linked glycan(s). We investigated the glycosylation of the 7.62 region containing epitope-surrounding sequences. The soluble 7.62 peptide (SKMep762) was purified on the basis of SKM9-2 reactivity (Fig. 4a). The purified SKMep762 was analyzed using liquid chromatography (LC)-mass spectrometry (MS) (electron-transfer dissociation (ETD)) after a lysyl endopeptidase (Lys-C)-digestion and desialylation. In terms of the major peaks in the LC analysis, the *N*-terminal (Fig. 4b) or *C*-terminal peptide (Fig. 4c) had three or two *O*-linked glycans that each contained a hexose and an *N*-acetylhexosamine, respectively. These results indicate that, in the SKM9-2 epitope region, three Ser/Thr residues (Ser^893^, Ser^900^, and Thr^903^), but not Ser^895^ and Ser^897^, are modified with *O*-linked glycans, which are probably core 1 or sialylated core 1 variants.

**Figure 5:**
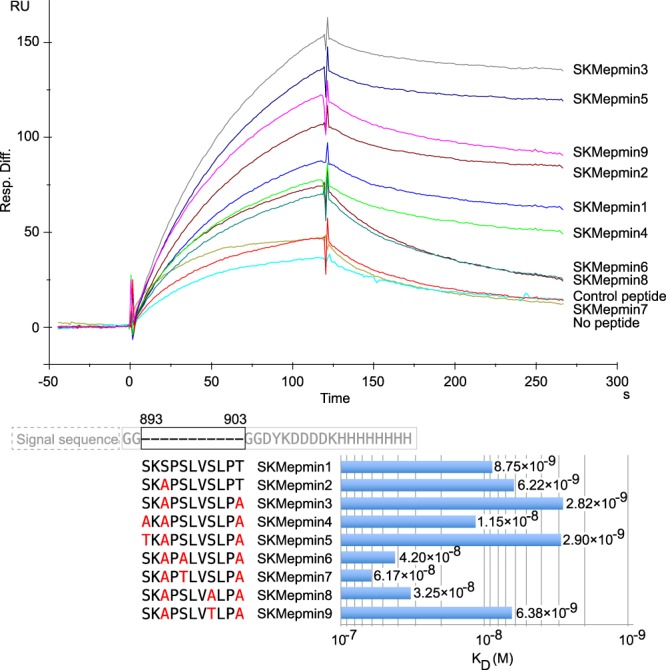
SPR analysis of the binding kinetics between SKM9-2 and mutants of minimum epitope. A soluble His-tagged mutant of minimum epitope (SKMepmin) was secreted from transfected HEK293T and immobilized as a ligand on the Ni^2+^-binding sensor chip NTA. The upper sensorgrams show the binding kinetics of analyte (5 µg/mL of SKM9-2) to SKMepmins. The control without any peptide is shown as “No peptide” and the negative control peptide cycle is shown as “Control peptide”. The glycan-digested SKMepmin3 that was treated with Neuraminidase A and *O*-Glycosidase was used as the negative control peptide. The dissociation constant in the lower bar graph was calculated using the manufacturer’s instructions. RU, response units; Signal sequence, the artificial signal sequence (MAPLLLLLLPLLLLPPAAP).

Since the position of sialylated glycan on SKMep762 could not be determined, we investigated the sialylation using minimum glycosylation peptides that could be bound by SKM9-2. The minimum glycosylation peptides were examined by surface plasmon resonance (SPR) analysis using soluble mutants of the minimum epitope (Fig. 5). Alanine substitutions for Ser^895^ and Thr^903^ increased the affinity to SKM9-2 primarily through incremental changes to the association rate constants (SKMepmin1, 2, and 3). Glycan attachments onto these residues may interfere with the binding of SKM9-2. Further alanine substitution for Ser^893^, Ser^897^, or Ser^900^ decreased the affinity of SKM9-2 in contrast to SKMepmin3 (SKMepmin4, 6, and 8). The substitution for Ser^897^, or Ser^900^ increased the dissociation rate (SKMepmin6 and 8). The SKM9-2 binding affinity was restored by threonine substitution at position 893 or 900 (SKMepmin5 and 9), whereas the binding ability was further reduced by the replacement of Ser^897^ with Thr (SKMepmin7). In conjunction with the results of alanine substitution, as shown in Figs 3, 5, SKM9-2 binding requires the *O*-linked glycan modification of Ser^893^ and Ser^900^ and non-glycosylated Ser^897^. Ser^895^ and Thr^903^ are not important for the SKM9-2 binding. Thus, SKMepmin3 could be used as the minimum glycosylation peptide.

**Figure 6:**
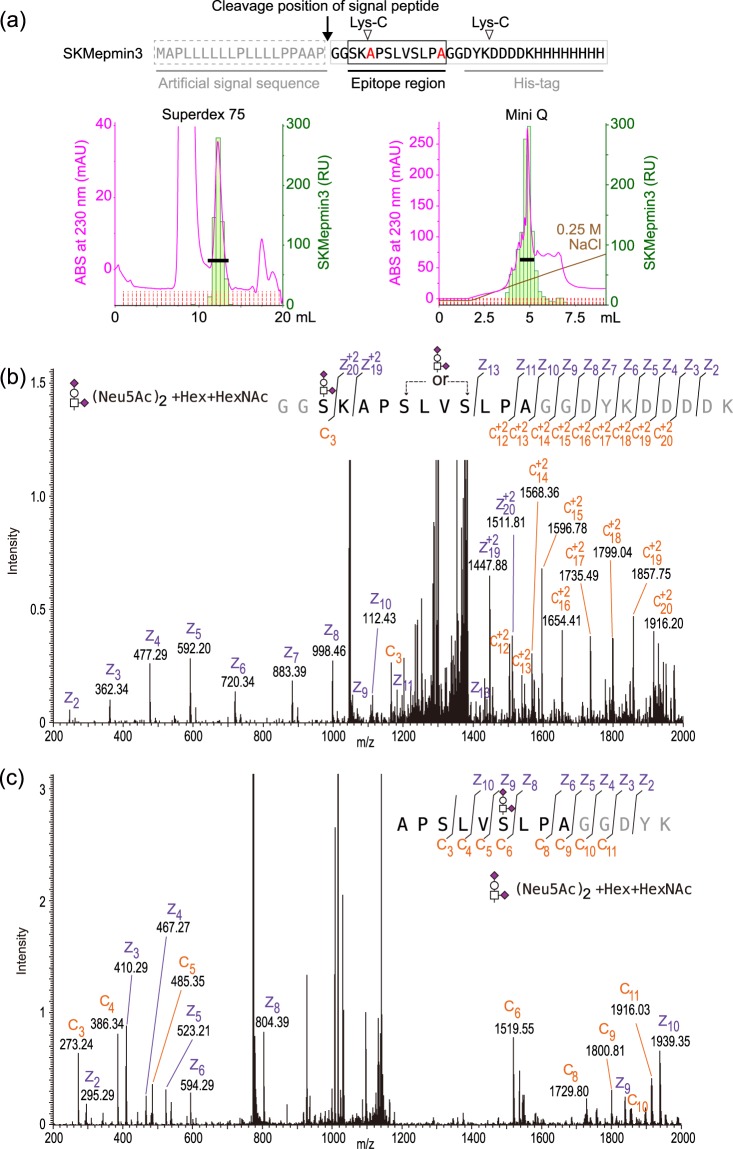
MS analysis of SKMepmin3. (**a**) Purification of SKMepmin3. Sequences of SKMepmin3 are shown in the top panel. The SKMepmin3 was purified using Ni-NTA column and SKM9-2-immobilized resins, and then separated by Superdex 75 10/300 GL (left chromatogram) and Mini Q 4.6/50 PE (right chromatogram). SKM9-2 binding activity was measured by SPR analysis. Pooled fractions are indicated with a black bar. (**b**) LC-MS spectrum of SKMepmin3 ion. Purified SKMepmin3 was partially digested with Lys-C and analyzed by LC-MS (ETD). MS/MS spectrum of a major component (*m/z* = 4186.74) in total ion current is shown. (**c**) LC-MS spectrum of digested SKMepmin3 ion. MS/MS spectrum of another major component (*m/z* = 2322.05) in total ion current is shown. Hex, hexose; HexNAc, *N*-acetylhexosamine; Neu5Ac, *N*-acetylneuraminic acid (sialic acid).

SKMepmin3 was purified through affinity purification using SKM9-2, size-exclusion chromatography, and anion exchange chromatography (Fig. 6a). An MS analysis of a major component suggested that the SKMepmin3 had two disialylated *O*-linked glycans. One was attached at Ser^893^ and the other was at Ser^897^ or Ser^900^ (Fig. 6b). The disialylated glycans consisted of two sialic acids, a hexose, and an *N*-acetylhexosamine. MS analysis of Lys-C-digested fragments indicated that the peptide had a disialylated *O*-linked glycan, containing two sialic acids, a hexose, and an *N*-acetylhexosamine, at Ser^900^ (Fig. 6c). These results concerning glycosylation position and the constituent of the core glycan were consistent with the results of SKMep762 analysis (Fig. 4).

**Figure 7:**
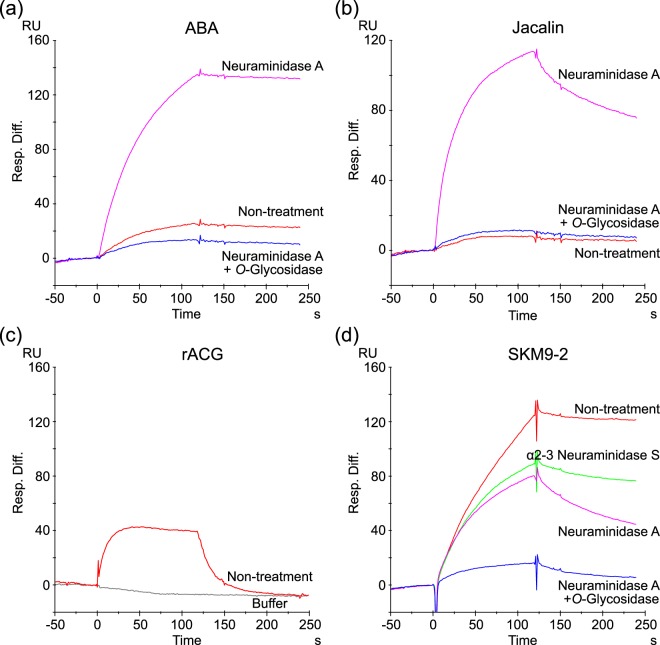
SPR analysis of bindings of lectin and SKM9-2 to glycosidase-treated SKMepmin3. Purified SKMepmin3 was incubated with α2-3 Neuraminidase S, Neuraminidase A, or Neuraminidase A + *O*-Glycosidase; and then was immobilized as a ligand on Ni^2+^-binding sensor chip NTA. The analytes were used at a concentration of 5 µg/mL. (**a**) ABA, (**b**) Jacalin, (**c**) rACG, (**d**) SKM9-2. Glycosidase-digested sample was not tested in the experiment with rACG.

Next, we investigated the structure of the glycan in SKMepmin3 by lectin binding analysis using SPR. As shown in Fig. 7a, *Agaricus bisporus* agglutinin (ABA) (a lectin that can bind to T antigen, monosialyl T, or disialyl T) bound to non-treated SKMepmin3 and its binding was enhanced by desialylation with Neuraminidase A that releases all sialic acid residues. ABA binding was lost after deglycosylation with *O*-Glycosidase and Neuraminidase A. Thus, we hypothesized that the glycan in SKMepmin3 is sialylated core 1 *O*-linked glycan. On the contrary, Jacalin (a lectin that binds to T antigen, α2-3 sialylated T antigen, but not disialyl T and α2-6 sialylated T containing α2-6 sialylated GalNAc^19^) did not bind to non-treated SKMepmin3. Jacalin recognized the desialylated SKMepmin3 with Neuraminidase A, but not the deglycosylated SKMepmin3 (Fig. 7b). These results suggest that SKMepmin3 has a core 1 glycan containing α2–6 sialylation. Besides, recombinant *Agrocybe cylindracea* lectin (rACG), an α2–3 sialic acid binder, interacted with the non-treated SKMepmin3 (Fig. 7c), suggesting that α2–3 sialylation would also be present in SKMepmin3. We also examined SKM9-2 binding to neuraminidase-treated SKMepmin3 (Fig. 7d). SKM9-2 binding was weakened by α2–3 Neuraminidase S, which is a highly specific exoglycosidase that cleaves α2,3-linked sialic acid, and the persistent binding of SKM9-2 was lost after desialylation with Neuraminidase A. The complete digest of glycan using *O*-Glycosidase eliminated the interaction between SKM9-2 and SKMepmin3 (Fig. 7d). These results suggest that SKMepmin3 contains terminal α2–3 sialylation and that α2–6 sialylation is required for the stable binding of SKM9-2. Based on the results shown in Figs 6, 7, we identified that the glycan in SKMepmin3 is disialyl T containing α2–6 sialylated GalNAc and α2,3-linked terminal sialic acid.

Taken together, the results suggest that SKM9-2 recognizes the peptide sequence of HEG1 (893-SKSPSLVSLPT-903) modified with disialyl T at the positions of Ser^893^ and Ser^900^, and that SKM9-2 does not bind to the peptide without glycosylation.

### Glycan of SKM9-2 epitope on mesothelioma cells

**Figure 8:**
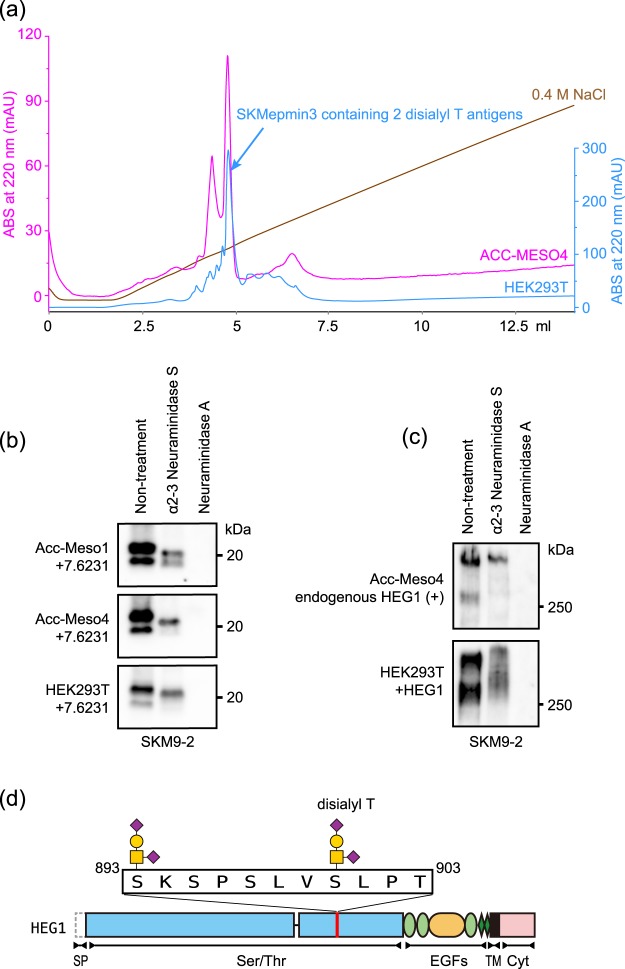
SKM9-2 epitope produced from mesothelioma cells. (**a**) Chromatogram of SKMepmin3 separation using anion exchange column. SKMepmin3 produced from HEK293T (blue line) or ACC-MESO4 (pink line) was affinity-purified using SKM9-2-immobilized resins and separated by Mini Q 4.6/50 PE. A major peak of SKMepmin3 purified from HEK293T contained two disialyl T antigens (Fig. 6). (**b**) Western blotting for epitope-fused GPI-anchor protein (7.6231) treated with neuraminidases. Cell lysate of 7.6231-transfected ACC-MESO1 (top panel), ACC-MESO4 (middle panel), or HEK293T (lower panel) was treated with α2-3 Neuraminidase S or Neuraminidase A, resolved by 4–15% SDS-PAGE, and analyzed by western blotting using SKM9-2. (**c**) Western blotting for full-length HEG1 treated with neuraminidases. Cell lysates of ACC-MESO4 (upper panel) and HEG1-transfected HEK293T (lower panel) were treated with α2-3 Neuraminidase S or Neuraminidase A and analyzed by 6% SDS-PAGE and western blotting using SKM9-2. (**d**) Schematic of SKM9-2 epitope. Full-length blots are presented in Supplementary Fig. S10.

The SKM9-2 epitope produced by mesothelioma cells was compared with the epitope produced in HEK293T cells. A major component of SKMepmin3 of ACC-MESO4 cells was eluted from an anion exchange column at the same retention volume as the SKMepimin3 of HEK293T cells containing two disialyl T antigens (Fig. 8a). Furthermore, SKM9-2 binding to the epitope-fused GPI-anchor protein (7.6231) on mesothelioma cells (ACC-MESO1 and ACC-MESO4) was completely lost after treatment with Neuraminidase A, while the binding was only partially decreased by α2–3 Neuraminidase S. Similar results were observed in 7.6231 on HEK293T (Fig. 8b). The results of the SKM9-2 reactivity to a neuraminidase-treated epitope on the membrane were consistent with those for the purified SKMepmin3 of HEK293T (Fig. 7d). Native endogenous HEG1 in ACC-MESO4 and recombinant HEG1 in HEK293T were also treated with neuraminidases. SKM9-2 binding was weakened by α2–3 Neuraminidase S and was lost by Neuraminidase A (Fig. 8c), consistent with the results of neuraminidase-treatment of 7.6231 (Fig. 8b). These results suggest that the SKM9-2 epitope of mesothelioma cells has disialyl T in the same way as SKMepmin3. Therefore, SKM9-2 would recognize the HEG1 peptide sequences (893-SKSPSLVSLPT-903) containing two disialyl T antigens at the positions of Ser^893^ and Ser^900^, but not Ser^897^, in mesothelioma cells as well (Fig. 8d).

## Discussion

mAb against an *O*-linked glycosylated region in a mucin-like protein is often an excellent tool for the detection of a disease. There are three types of such mAbs. [1] mAb against aberrant glycan clusters. In cell proliferative diseases such as tumors, immature *O*-linked glycans, T antigen, and Tn antigen with/without sialylation, are attached in clusters on mucin or mucin-like proteins. mAb recognizing these glycan clusters are used as tumor markers^20^. [2] mAb against aberrant mucin in which the core peptide is not sufficiently modified by glycans. An alteration in carbohydrate chain synthesis in the tumor cells can cause the expression of an aberrant mucin, wherein the core peptide is not sufficiently modified by *O*-linked glycans. The aberrant mucin with hypoglycosylation can become a tumor antigen^21,22^. [3] mAb against atypically glycosylated amino acid sequences. A mAb recognizing *O*-linked glycosylated amino acid sequences of a mucin-like protein is useful for detecting an antigen produced by abnormal cells^11,23,24^. These mAbs, such as LpMab-12^23^, UN1^24^, and KL-6^25^, can bind to peptides containing several types of glycans; i.e., they may recognize the conformational structure of amino acid residues that is affected by glycosylation rather than the structure of both the amino acid sequences and the attached glycan. Such glycopeptide-recognizing mAb has been used in clinical trials of antibody therapeutics against cancer. Recently, the therapeutic effect of antibody-drug conjugate (ADC) or chimeric antigen receptors (CAR) using glycopeptide-recognizing mAb has been investigated clinically^26^.

SKM9-2 was a “type 3” mAb, recognizing the *O*-linked glycosylated amino acid sequences of HEG1. SKM9-2 could recognize the epitope containing several variations of sialylation. SKMepmin3 affinity-purified by SKM9-2 was fractionated as multiple peaks with a range of binding affinities on ion-exchange chromatography (Mini Q in Fig. 6a and Supplementary Fig. S1). The product of the major peak, showing high SKM9-2 binding activity and quantity, was identified as a peptide containing two disialyl T antigens at the positions of Ser^893^ and Ser^900^ (Fig. 6). The product of an earlier eluting peak would potentially be modified with glycan(s) containing less sialylation, since it was less negatively charged and was bound by Jacalin, which does not recognize disialyl T (Supplementary Fig. S1). These results suggest that SKM9-2 does not have a strict recognition for the number and/or linkage of sialylation. In fact, SKM9-2 could interact with SKMepmin3 containing desialylated core 1, although its dissociation rate was high (Neuraminidase A in Fig. 7d). Products of the later eluting peak in the ion-exchange chromatogram were more negatively charged and had weaker SKM9-2 binding activity than the major peak product (Mini Q in Fig. 6a). The later eluting peak products might potentially contain a different glycan, containing at least four sialic acids and/or another negatively charged modification, such as sulfation. Such glycosylations in the epitope may influence SKM9-2 binding, therefore we will investigate glycan modifications that influence the SKM9-2 binding in future study. Sialyl Lewis^X^, which is detected in HEG1^15^, was not present in the SKM9-2 epitope, since the glycan in SKMepmin3 was not bound by an anti-sialyl Lewis^X^ antibody (Supplementary Fig. S2).

In this study, we analyzed the epitope peptides that are produced by human cell lines in serum-free culture media. The sialic acid of the epitope peptide was detected using higher-energy collisional dissociation (HCD) MS spectra and was identified as *N*-acetylneuraminic acid (Neu5Ac) without *N*-glycolylneuraminic acid^27^ (Supplementary Fig. S3). SKM9-2 recognizes the HEG1 on the human cells^15^. Therefore, almost sialic acids of the epitope in human mesothelioma would be Neu5Ac. However, *N*-glycolylneuraminic acid, which cannot be synthesized in humans, and *O*-acetylated sialic acids are often detected in tumor and mucinous tissues^28–32^. These sialic acid derivatives in *in vivo* tissues may influence the sensitivity and specificity of mesothelioma detection. Although there were no apparent differences in SKM9-2 immunostaining among *in vitro* cultured cell lines, *in vivo* xenografted cell lines, and the mesothelioma specimen (Supplementary Fig. S4), detailed analyses of the sialic acid derivatives may be necessary in a future study.

The substitution of threonine for Ser^900^ increased the dissociation rate between SKM9-2 and its epitope peptide (SKMepmin9 in Fig. 5). This demonstrates that a minor variation at position 900, introduced by the addition of a methyl group, could affect the persistent binding of SKM9-2. It has been reported that the attached carbohydrate moiety adopts a different orientation on serine and threonine^33^, and the difference between serine and threonine affects glycan-recognition with lectin^34^ and binding of anti-MUC1 antibody (SM3)^35^. Thus, the structure at position 900, containing the orientation of glycan, may concern epitope formation directly. On the contrary, threonine substitution for Ser^893^ hardly influenced the association and dissociation rates (SKMepmin5 in Fig. 5), while alanine substitution decreased the association rate (SKMepmin4 in Fig. 5). The glycan at position 893 may increase SKM9-2 binding by promoting the conformational change of the surrounding amino acid residues. The S893A mutant of 7.6231 was not recognized by SKM9-2 as seen in the western blot (Fig. 3a). In contrast, SKMepmin4 had a similar dissociation constant to SKMepmin1 without substitution, despite the same alanine substitution for Ser^893^ (Fig. 5). This discrepancy may be due to two more substitutions, S895A and T903A, in SKMepmin4. SKMepmin3, containing S895A and T903A, can bind to SKM9-2 more strongly than SKMepmin1 (Fig. 5). The alanine substitution at positions 895 and 903 may induce the conformational change of amino acid residues in the epitope, as well as the glycan modification at position 893.

It was difficult to identify the sialylated *O*-linked glycan in the epitope of native HEG1 on mesothelioma cells because the epitope peptides of endopeptidase-digested native HEG1 were not be obtained. The digested peptides were not captured by SKM9-2 (Supplementary Fig. S5). This may be due to protease resistance by the surrounding glycans and/or loss of antigen activity by cleavage at Lys^894^ in the epitope. Nevertheless, we think that the major glycan in the epitope of mesothelioma HEG1 would be disialyl T, since SKMepmin3 produced from mesothelioma cells showed similar characteristics to the purified SKMepmin3 containing disialyl T with respect to surface charge properties (Fig. 8a). The results of neuraminidase treatments of HEG1 or the epitope-fused protein on mesothelioma cells were also similar to those obtained from HEK293T (Fig. 8b,c).

The glycan of Thr^903^ may be larger than those on the other glycosylation sites because the molecular size of T903A mutant was clearly smaller than the size of the other mutants (Fig. 3a). This position is predicted to be an *O*-glycosylation site with the polypeptide *N*-acetylgalactosaminyltransferase (ppGalNAcT) by two *O*-glycosylation prediction tools. The glycosylation prediction tool NetOGly 4.0^36^ showed a high prediction confidence score of 0.93925. Similarly, ISOGlyP^37^, which predictively calculates specific enhancement value of glycosylation with some ppGalNAcTs, also showed a high Enhancement Value Product: ppGalNAcT-1, 3.46; G ppGalNAcT-2, 4.28; ppGalNAcT-11, 4.45. However, this glycosylation was not required for the recognition of SKM9-2. The substitution of alanine for Thr^903^ did not suppress SKM9-2 binding (Fig. 3a); rather, the substitution increased the association rate (SKMepmin2 and 3 in Fig. 5a). Further elongation of the glycan at Thr^903^ could potentially interfere with the approach of SKM9-2 to the epitope in HEG1 and may influence the detection of mesothelioma with SKM9-2. The large glycan around the epitope, which might interfere with SKM9-2 binding, should be investigated on histological subtypes of mesothelioma in subsequent studies.

## Experimental Procedures

### Cell lines

Human malignant mesothelioma cell lines, ACC-MESO-1 (RCB2292) and ACC-MESO-4 (RCB2293)^38^, were obtained from the RIKEN Cell Bank (Tsukuba, Japan). Human embryonic kidney cell line HEK293T (RCB2202) was obtained from the RIKEN Cell Bank. The cell line 293 H was obtained from Thermo Fisher Scientific (Rockford, IL, USA). The mesothelioma cell lines and HEK293T cells were cultured in RPMI 1640 and Dulbecco’s Modified Eagle’s Medium, containing 10% fetal bovine serum, respectively. The 293 H cells expressing HEG1 peptides were cultured in 293 SFM II (Thermo Fisher Scientific).

### Cloning of HEG1 and the production of recombinant HEG1

Sequences of tested HEG1 are shown in Supplementary Dataset. Full-length HEG1 cDNA was synthesized from total RNA purified with TRIzol reagent (Thermo Fisher Scientific) from ACC-MESO-4, using an oligo dT primer and SuperScript II RNase H reverse transcriptase (Thermo Fisher Scientific). cDNA encoding the HEG1 open reading frame was amplified by PCR using PrimeSTAR HS DNA Polymerase (Takara Bio Inc., Shiga, Japan). The HEG1 cDNA was cloned and inserted into the pcDNA3.1(−) mammalian expression vector (Thermo Fisher Scientific). For analyses of *N*-terminal truncation, we inserted mutants into pFLAG-CMV1 (Sigma-Aldrich Japan K.K., Tokyo, Japan). For analyses of *C*-terminal deletion lacking the transmembrane domain, we used mutants that were fused with a GPI-anchor protein, SLURP1-gpi^18^, in pcDNA3.1(+) (Thermo Fisher Scientific) (Supplementary Dataset). Alanine substituted mutants were prepared using site directed mutagenesis. To produce a soluble epitope peptide, a modified HEG1 signal peptide and a *C*-terminal tag containing the FLAG tag plus a polyhistidine sequence were used in the mammalian expression vector pEF-BOS^39^ (Supplementary Dataset). The plasmid constructs were transfected with Lipofectamine 3000 (Thermo Fisher Scientific). The HEG1 peptide-expressing 293 H clone was obtained by selection with geneticin (Thermo Fisher Scientific).

### Western blotting

Monolayer cells were solubilized with 20 mM Tris buffer (pH 8.0) containing 1% SDS, 1 mM phenylmethylsulfonyl fluoride, 125 mU/mL Benzonase Nuclease (Merck Millipore Co., Tokyo, Japan) (25 µL/cm^2^ culture surface area). The lysate (10 µL/lane) was resolved by SDS-PAGE under reducing conditions using 6% polyacrylamide gels or 4–15% precast gels (Bio-Rad Laboratories Inc., Hercules, CA, USA). Then it was transferred to a polyvinylidene difluoride membrane (Immobilon-P; Merck Millipore Co.) by semi-dry blotting in a buffer containing 25 mM Tris, 192 mM glycine, 0.1% SDS, and 20% methanol. The membrane was blocked with 5% non-fat milk in 20 mM Tris-buffered saline (pH 7.2) containing 0.1% Tween 20 and reacted with 1 µg/mL of mAb SKM9-2 or anti-FLAG (Anti-DYKDDDDK tag mAb; 1E6, Wako Pure Chemical Industries, Osaka, Japan). After washing, it was treated with horseradish peroxidase-conjugated goat anti-mouse IgG (Jackson ImmunoResearch, West Grove, PA, USA), and developed with Amersham ECL select (GE Healthcare, Buckinghamshire, UK). Although glycan in non-fat milk often interferes the glycan-dependent binding of an antibody, the SKM9-2 binding was not affected by the blocking using non-fat milk, in comparison with 5% bovine serum albumin (Supplementary Fig. S6).

### Purification of SKMep762

A His-tagged epitope peptide (SKMep762, Supplementary Dataset) was secreted by 293 H cells stably expressing SKMep762. Culture supernatant (~300 mL) was applied to HisTrap excel (1 mL) (GE Healthcare) and the column was washed with 25 mM phosphate buffer (pH 7.2) plus 0.02% Tween 20 (PBT), containing 2 M (NH_4_)_2_SO_4_ and 0.5 M NaCl. The sample was eluted with PBT containing 0.5 M imidazole, 2 M (NH_4_)_2_SO_4_, and 0.5 M NaCl, passed through Resource Phe (1 mL) (GE Healthcare). Then 10 mM EDTA was added, and it was dialyzed against 20 mM Tris buffer (pH 8.0) containing 0.02% Tween 20 (TBT), using Spectra/Por 3 dialysis tubing (Spectrum Laboratories, CA, USA). It was further isolated by anion exchange chromatography using a Mono Q 5/50 GL column (GE Healthcare) connected to an AKTAexplorer 10 S (GE Healthcare). The peptide was eluted with a linear gradient of 0–0.5 M NaCl in TBT and was detected by dot blot analysis using SKM9-2. The pooled fraction was concentrated to 0.5 mL by Amicon Ultra-15 3 K devices (Merck Millipore Co.) and separated by Superdex 75 10/300 GL (GE Healthcare) in PBT containing 0.5 M NaCl. Fractions #22–23 (Superdex 75 in Fig. 4a) were pooled, dialyzed against TBT, and isolated by anion exchange chromatography using a Mini Q 4.6/50 PE column (GE Healthcare). The peptide eluted in fractions #25–29 (Mini Q in Fig. 4a) with a linear gradient of 0–0.5 M NaCl in 20 mM Tris buffer (pH 8.0). The pooled fraction was dialyzed against water and its UV absorbance was measured at 215 and 225 nm. The peptide concentration was quantified by UV absorbance using the formula: concentration (µg/mL) = (A215 nm − A225 nm) × 144^40^.

### Purification of SKMepmin3

HEK293T cells were transfected with plasmid constructs of a His-tagged epitope peptide (SKMepmin3, Supplementary Dataset) and cultured for 72 h in 293 SFM II. Culture supernatant (~300 mL) was applied to Ni-NTA Cartridge (5 mL) (Wako Pure Chemical Industries) and the column was washed with PBT containing 0.5 M NaCl. The sample was eluted with PBT containing 0.5 M imidazole and 0.15 M NaCl, and incubated at 4 °C for 18 h with SKM9-2-binding protein G-Sepharose (300 µg of SKM9-2 per 350 µL gel). The gels were collected by centrifugation and the peptides were eluted with TBT containing 1 mM EDTA and 6 M guanidine hydrochloride. The eluate (2 mL) was concentrated to 0.5 mL by Amicon Ultra-15 3 K devices and separated by Superdex 75 10/300 GL in the same buffer. The SKM9-2-binding peptides were detected by SPR using Biacore 3000 (GE Healthcare). The fractions #21–25 (Superdex 75 in Fig. 6a) were pooled, dialyzed against TBT using Spectra/Por 3 dialysis tubing, and isolated by anion exchange chromatography using a Mini Q 4.6/50 PE column. The peptide eluted in fractions #19–35 with a linear gradient of 0–0.5 M NaCl in 20 mM Tris buffer (pH 8.0). Pooled fractions (#23–26) (Mini Q in Fig. 6a) were dialyzed against water.

### LC-MS analysis

The purified peptides were digested with Lys-C (Wako Pure Chemical Industries) in 50 mM Tris buffer (pH 8.6) at 37 °C for 5 h. For SKMep762, the digest was acidified with TFA to pH 2.0 and incubated at 80 °C for 2 h for desialylation. The peptide sample was analyzed by LC-MS as described previously^41^ with slight modifications. Briefly, the digest was acidified with 0.2% formic acid and loaded on a trap column (Monocap C18 for trap, 0.2 mm id × 50 mm; GL-Science Inc., Tokyo, Japan) at 0.015 mL/min. After washing with 0.1% formic acid, the column was connected to a nanoflow LC system. The peptides were separated on a tip column (C18 column, 0.15 mm id × 100 mm, 3 µm particles; Nikkyo Technos Co., Ltd, Tokyo, Japan) with 5–35% acetonitrile gradient (70 min) in 0.1% formic acid at flow rate of 200 nL/min. The effluent was ionized by electrospray and introduced directly into a mass spectrometer (LTQ Orbitrap Velos ETD, Thermo Fisher Scientific) with a source voltage of 2.0 kV and a capillary temperature of 250 °C. The mass spectrometer was operated by data-dependent acquisition in positive mode. The MS1 was obtained with an Orbitrap analyzer at a resolution of 30,000 at m/z 400 (scan range: m/z 300–2000, locked at 445.120030). ETD MS2 data for the most intense single signal was obtained with an iontrap analyzer at an activation time of 0.1 sec, microscan of 5, and exclusion of 60 s. In case of SKMepmin3, HCD MS2 spectra were also acquired with Orbitrap (resolution: 7500, NCE: 35) as well as ETD spectra (with ion trap). The spectra were analyzed with Xcalibur (ver. 2.2, Thermo Fisher Scientific). Assignment of signals on ETD MS2 spectra was performed manually, based on the information of fragment ion mass predicted using a web application, Protein Prospector, ver. 5.22.0 (http://prospector.ucsf.edu/prospector/mshome.htm).

### Deglycosylation analysis

Cell lysate or SKMepmin3 was treated with α2-3 Neuraminidase S, α2-3, 6, 8, 9 Neuraminidase A, and *O*-Glycosidase (New England Biolabs Japan, Tokyo, Japan) in 50 mM sodium phosphate (pH 7.5) containing 1% Nonidet P-40, according to the manufacturer’s instructions. The cell lysate sample was resolved by 4–15% or 6% SDS-PAGE under reducing conditions and detected by western blotting using SKM9-2. The analysis of the SKMepmin3 sample was performed by SPR.

### SPR analysis

The binding of SKM9-2 or lectin to SKMepmin was measured by SPR analysis. SKMepmin was diluted with a running buffer (10 mM HEPES buffer (pH 8.1) containing 150 mM NaCl and 0.005% Tween-20), flowed on Ni^2+^-binding sensor chip NTA (GE Healthcare), and affinity-bound through His-tag as a ligand on the sensor chip. Binding analysis of SKM9-2 was performed in a running buffer containing 50 µM EDTA. A non-treated flow cell was used as a reference cell. SKM9-2, anti-sialyl Lewis^X^ (CSLEX1, Becton, Dickinson and Company Japan, Tokyo, Japan), rACG (Wako Pure Chemical Industries), biotinylated ABA (J-Oil Mills, Inc., Tokyo, Japan), or biotinylated Jacalin (Vector Laboratories, Inc., Burlingame, CA, USA) (each 5 µg/mL) was injected as an analyte with the running buffer. As a regeneration buffer, 350 mM EDTA was used. The dissociation constant was calculated using the manufacturer’s instructions.

## Electronic supplementary material

Supplementary Figures

Supplementary Dataset

## Data Availability

All data generated or analyzed during this study are included in this published article and its Supplementary Information files.
