# Supplementary material for: Identification of mesothelioma-specific sialylated epitope recognized with monoclonal antibody SKM9-2 in a mucin-like membrane protein HEG1
Source: Sci Rep. 2018 Sep 24;8:14251. doi: 10.1038/s41598-018-32534-8 (PMC6155162; doi:10.1038/s41598-018-32534-8)
Supplement: Supplementary file 1 — Supplementary Figures [file 41598_2018_32534_MOESM1_ESM.pdf]

Supplementary Information

Identification of mesothelioma-specific sialylated epitope recognized with monoclonal antibody SKM9-2 in a mucin-like membrane protein HEG1

Rieko Matsuura[1], Hiroyuki Kaji[2], Azusa Tomioka[2], Takashi Sato[2], Hisashi Narimatsu[2], Yasuhiro Moriwaki[3], Hidemi Misawa[3], Kohzoh Imai[4], and Shoutaro Tsuji[1]*.

[1]Kanagawa Cancer Center Research Institute, Yokohama, Japan; [2]Glycoscience & Glycotechnology Research Group, Biotechnology Research Institute for Drug Discovery, National Institute of Advanced Industrial Science and Technology, Tsukuba, Japan; [3]Division of Pharmacology, Faculty of Pharmacy, Keio University, Tokyo, Japan; [4]Institute of Medical Science, University of Tokyo, Tokyo, Japan

* Address correspondence to: Shoutaro Tsuji, Ph.D.: Kanagawa Cancer Center Research Institute, 2-3-2 Nakao, Asahi-ku, Yokohama-shi, Kanagawa 241-8515, Japan; Phone: +81-45-520-2222 ext. 4037; Fax: +81-45-520-2216; E-mail: stsuji@gancen.asahi.yokohama.jp

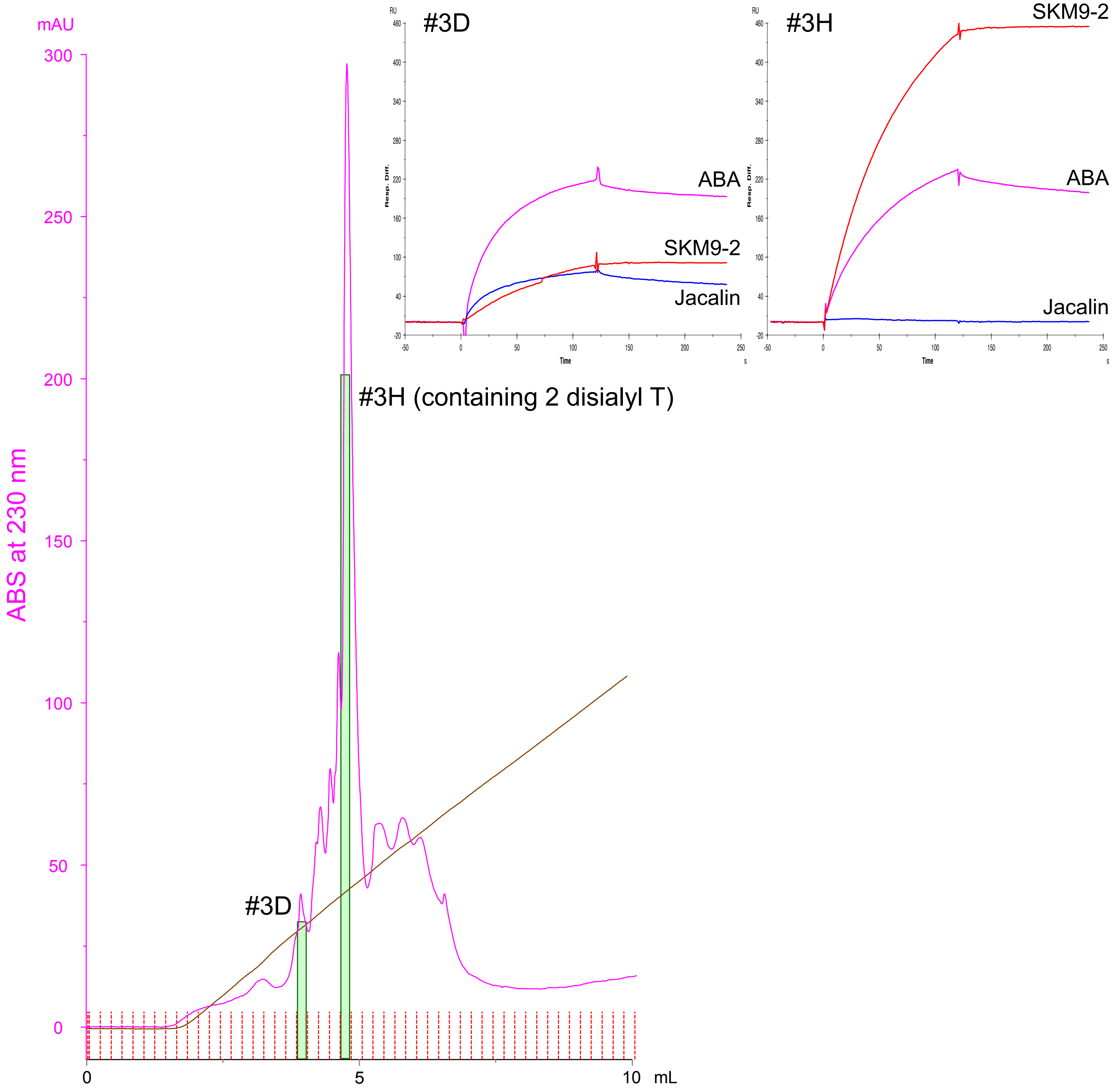

Supplementary Fig. S1. Mini Q separation and lectin binding of SKMepmin3 produced by HEK293T. The binding activities of fractionated peaks (#3D and #3H) to SKM9-2, ABA, and Jacalin were measured by SPR analysis. Fraction (5-fold dilution) was immobilized as a ligand on Ni^{2+}-binding sensor chip NTA. The analytes were used at the concentration of 5 μg/mL.

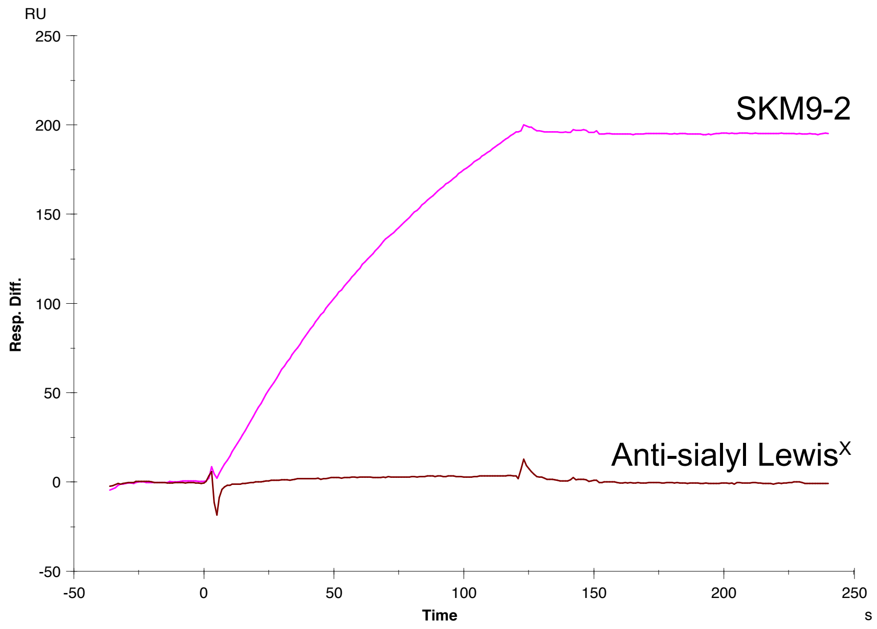

Supplementary Fig. S2. SPR analysis of binding of anti-sialyl Lewis^X to SKMepmin3. Purified SKMepmin3 was immobilized as a ligand on Ni^{2+}-binding sensor chip NTA. The analytes were used at the concentration of 5 μg/mL. Anti-sialyl Lewis^X did not bind to SKMepmin3.

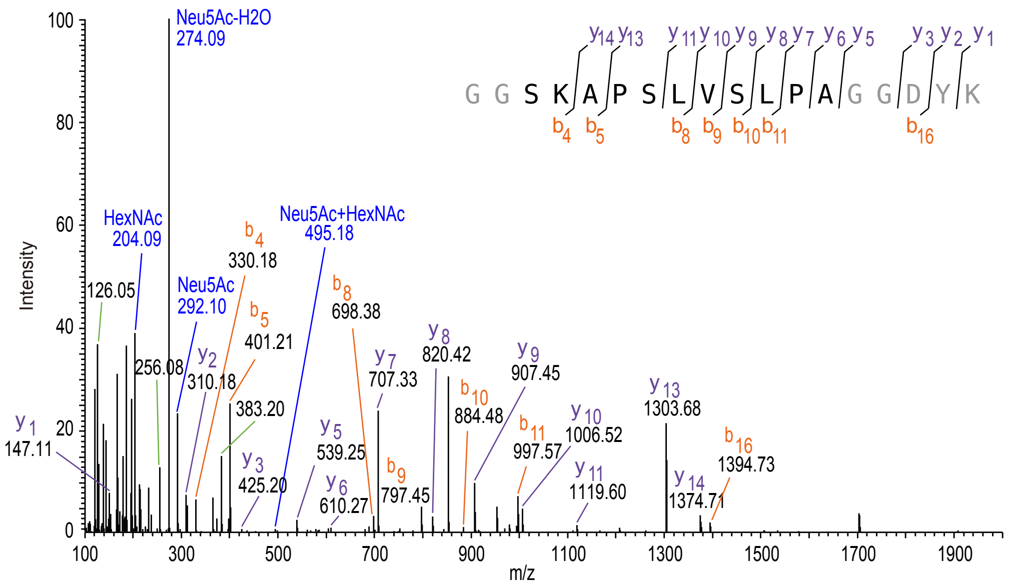

Supplementary Fig. S3. MS analysis of sialic acid in SKMepmin3.

LC-MS spectrum of SKMepmin3 ion. Purified SKMepmin3 was partially digested with Lys-C and analyzed by LC-MS (HCD). MS/MS spectrum of a component (*m/z* = 3598.54) in total ion current is shown. HexNAc, *N*-acetylhexosamine; Neu5Ac, *N*-acetylneuraminic acid (sialic acid).

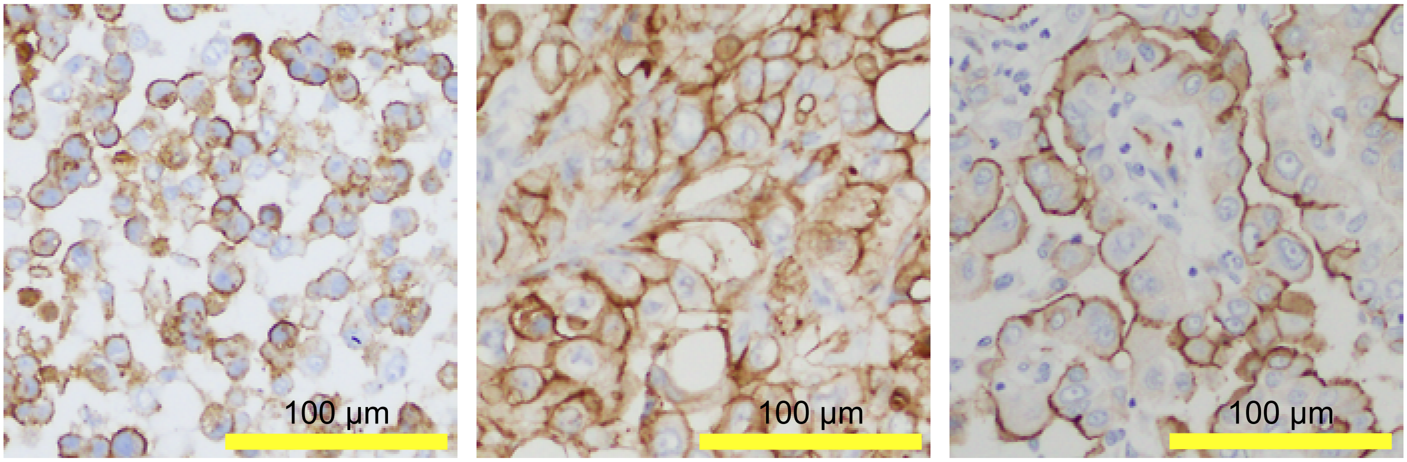

ACC-MESO4
In virto cultured cells

ACC-MESO4
In vivo xenografted cells

Mesothelioma tissue

Supplementary Fig. S4. Immunohistochemical staining of mesothelioma cells using SKM9-2. *In vitro* cultured cells (ACC-MESO4) were centrifuged and the precipitate was fixed with formalin. The cell block was embedded in paraffin. *In vivo* xenograft tissues were obtained from ICR-nu mice (Charles River Laboratories International, Inc., Kanagawa, Japan) that were injected intradermally with ACC-MESO4. Animal experiment was approved and performed in accordance with the animal care and use committees of Kanagawa Cancer Center. Mesothelioma tissue was obtained from US Biomax, Inc. (Rockville, MD, USA) as a Tissue microarray (T392a).
The sections were immunostained as described previously (15). Scale bars, 100 µm.

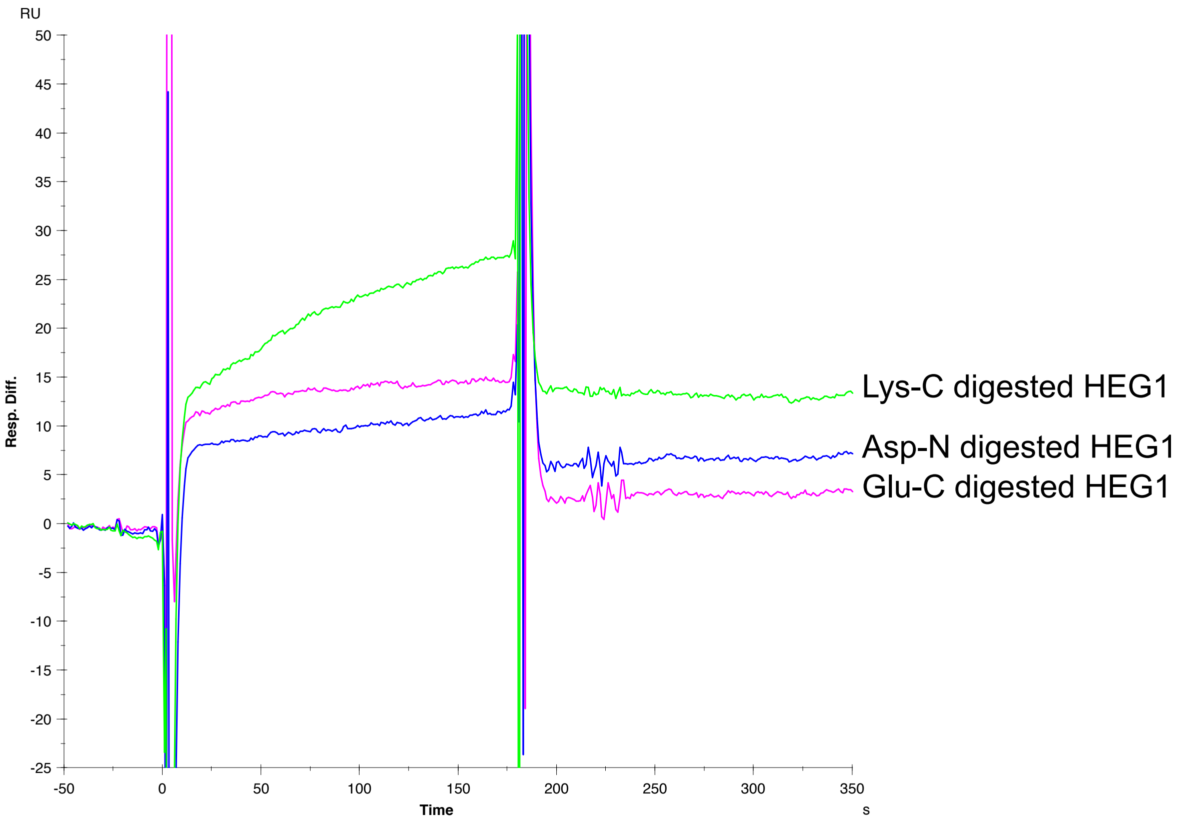

Supplementary Fig. S5. Binding of endopeptidase-digested native HEG1 to SKM9-2. Native HEG1 purified from ACC-MESO4 (15) was digested with endopeptidases (Roche Diagnostics GmbH, Mannheim, Germany). The reaction of samples was stopped with EDTA and phenylmethanesulfonyl fluoride. The SKM9-2 binding of digested peptides was tested by Biacore using SKM9-2-captured anti-mouse Ig-immobilized sensor chip.

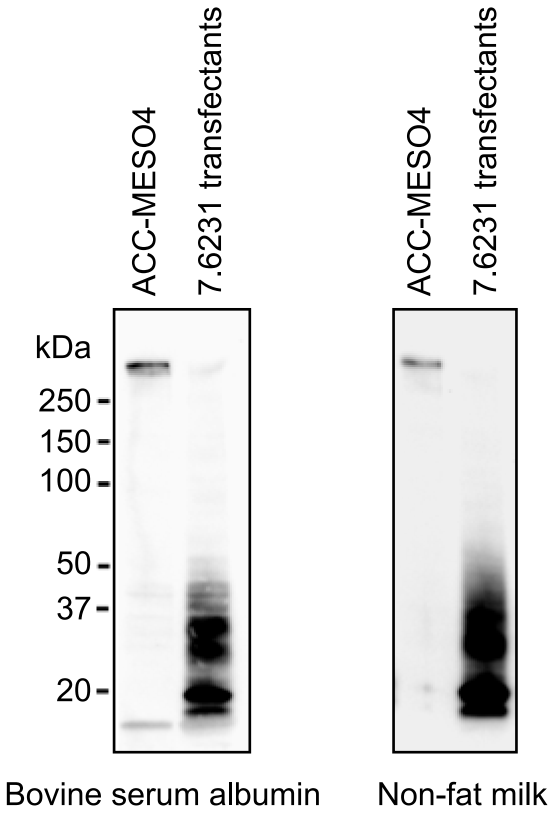

Supplementary Fig. S6. Western blotting using SKM9-2 under the different blocking agents. As blocking agent, 5% non-fat milk or 5% bovine serum albumin were used in 20 mM Tris-buffered saline (pH 7.2) containing 0.1% Tween 20. The blocking with bovine serum albumin was done for 18 h at room temperature. Cell lysate samples were resolved by 4–15% SDS-PAGE and analyzed by western blotting using SKM9-2.

in Fig. 1

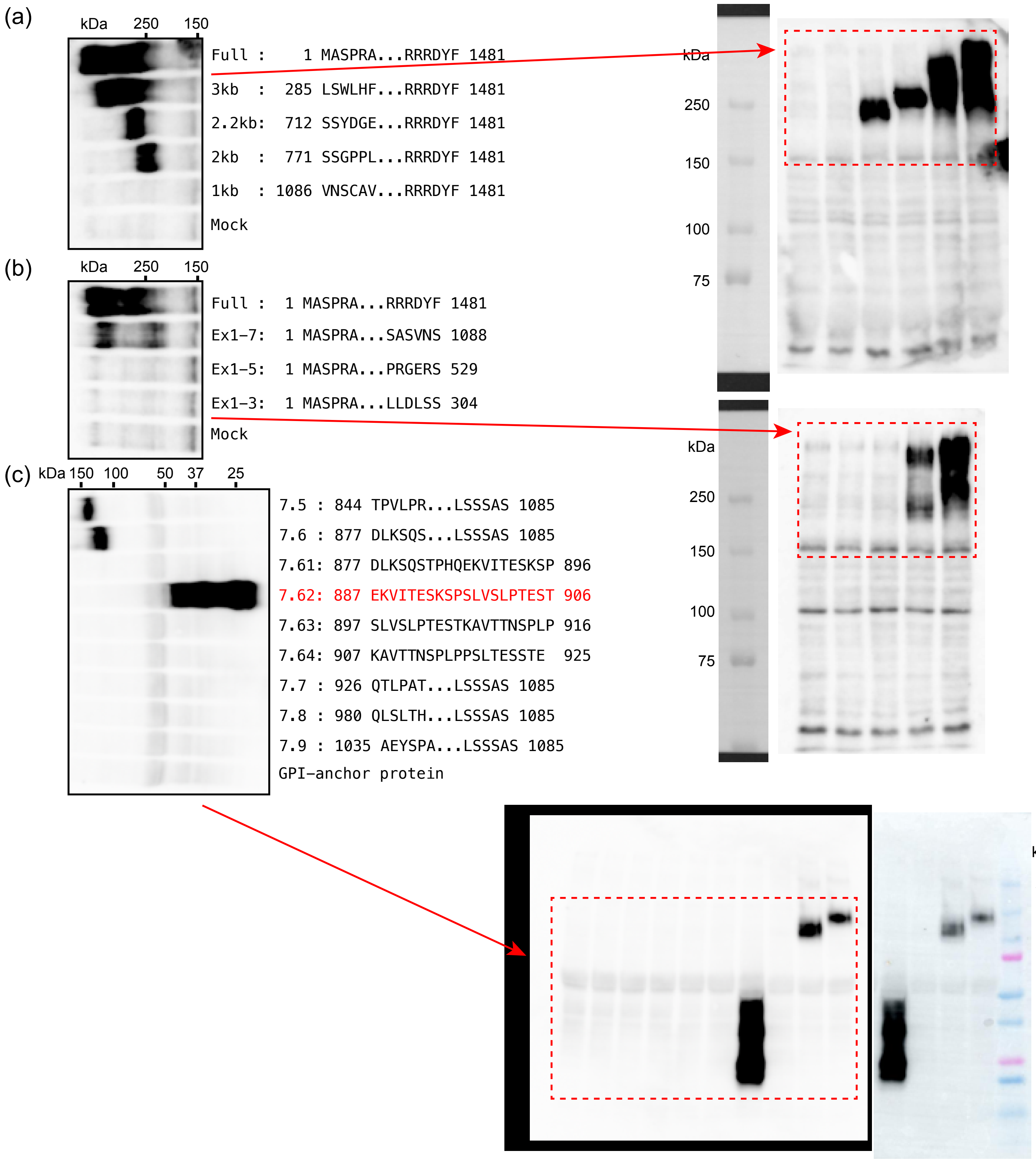

Supplementary Fig. S7. Full-length western blots of Figure 1.

Red dotted boxes show the cropping locations.

in Fig. 2

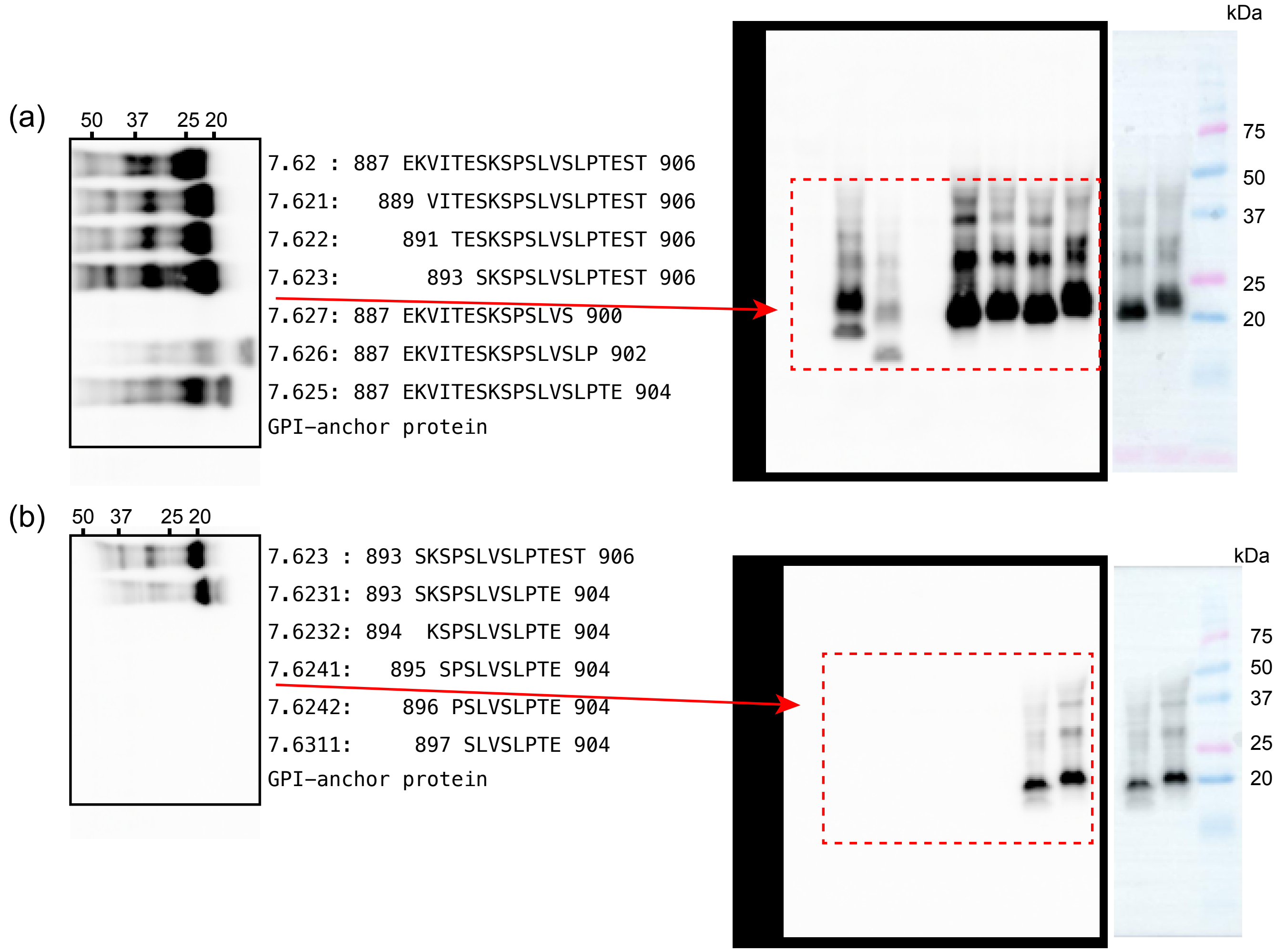

Supplementary Fig. S8. Full-length western blots of Figure 2.

Red dotted boxes show the cropping locations.

in Fig. 3

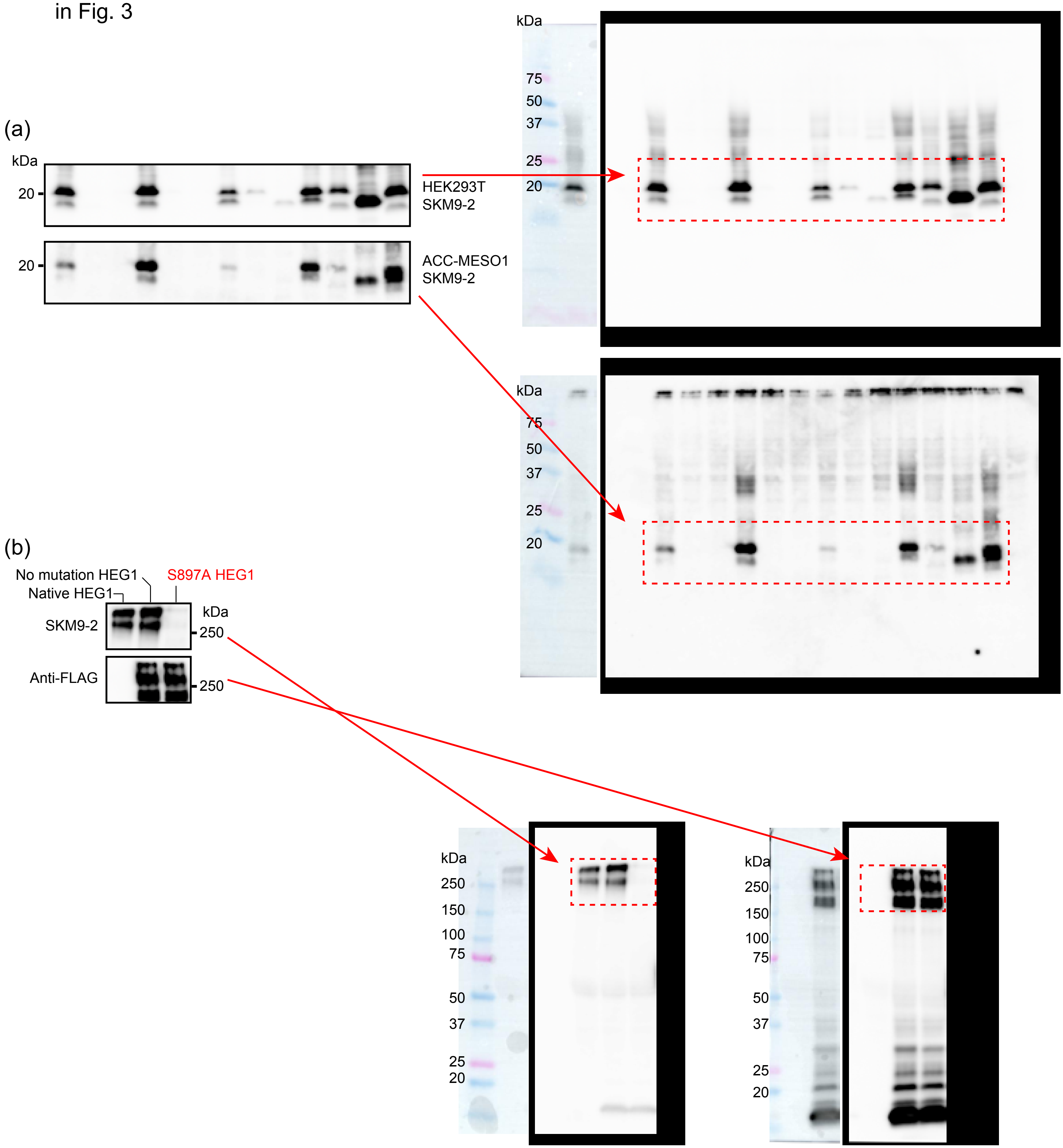

Supplementary Fig. S9. Full-length western blots of Figure 3.

Red dotted boxes show the cropping locations.

in Fig. 8

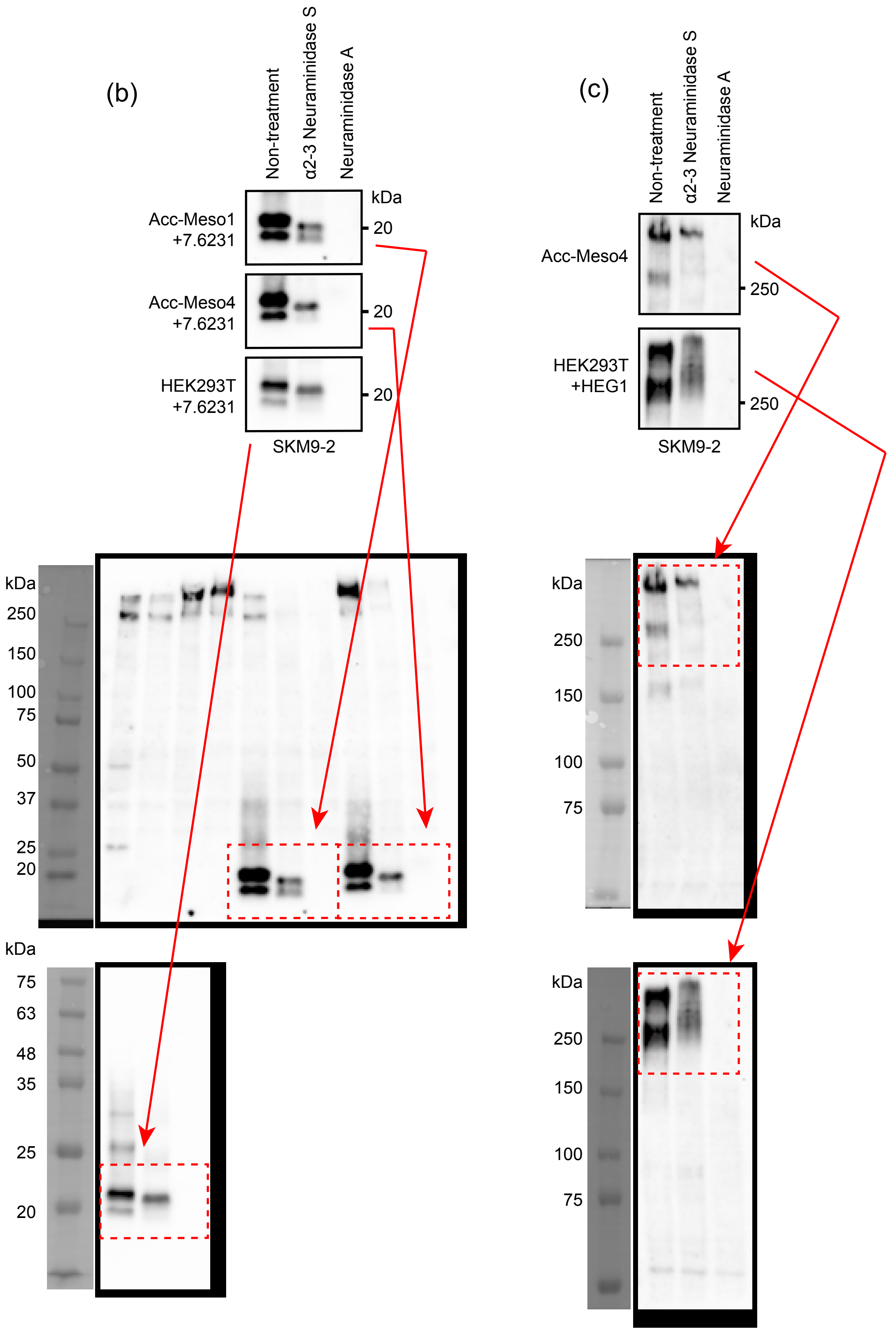

Supplementary Fig. S10. Full-length western blots of Figure 8.

Red dotted boxes show the cropping locations.
